# Synergistic Effects of Calcium Phosphate Biomaterials Combined with Honey on Osteochondral Regeneration: A Qualitative Study in an Animal Model

**DOI:** 10.3390/bioengineering13050585

**Published:** 2026-05-20

**Authors:** Pavol Rusnák, Katarína Vdoviaková, Ján Danko, Lenka Krešáková, Filip Humeník, Ľubomír Medvecký, Mária Giretová, Radoslava Štulajterová, Kristína Čurgali, Štefan Tóth, Jozef Bíreš, Filip Korim, Zuzana Čriepoková, Peter Očenáš, Roman Totkovič, Tatiana Špakovská

**Affiliations:** 1 Hospital AGEL Košice-Šaca, Lúčna 57, 040 15 Košice-Šaca, Slovakia; pavol.rusnak@nke.agel.sk (P.R.); roman.totkovic@nke.agel.sk (R.T.); tatiana.spakovska@nke.agel.sk (T.Š.); 2 Department of Morphological Disciplines, University of Veterinary Medicine and Pharmacy in Košice, Komenského 73, 041 81 Košice, Slovakia; jan.danko@uvlf.sk (J.D.); lenka.kresakova@uvlf.sk (L.K.); filip.humenik@uvlf.sk (F.H.); filip.korim@student.uvlf.sk (F.K.); 3 Division of Functional and Hybrid Systems, Institute of Materials Research of SAS, Watsonova 47, 040 01 Košice, Slovakia; lmedvecky@saske.sk (Ľ.M.); mgiretova@saske.sk (M.G.); rstulajterova@saske.sk (R.Š.); 4 Department of Histology and Embryology, Faculty of Medicine, Pavol Jozef Šafárik University in Košice, Trieda SNP 1, 040 11 Košice, Slovakia; kristina.curgali@upjs.sk (K.Č.); stefan.toth@upjs.sk (Š.T.); 5 Clinic of Ruminants, University of Veterinary Medicine and Pharmacy in Košice, Komenského 73, 041 81 Košice, Slovakia; jozef.bires@uvlf.sk; 6 Clinic of Horses, University of Veterinary Medicine and Pharmacy in Košice, Komenského 73, 041 81 Košice, Slovakia; zuzana.criepokova@uvlf.sk; 7 Department of Chemistry, Biochemistry and Biophysics, University of Veterinary Medicine and Pharmacy in Košice, Komenského 73, 041 81 Košice, Slovakia; peter.ocenas@uvlf.sk

**Keywords:** cartilage, calcium phosphates, manuka honey, regenerative potential, pig, model, scaffold

## Abstract

Osteochondral defects of the knee represent a significant clinical challenge due to the limited regenerative capacity of the osteochondral unit. The aim of this study was to evaluate the therapeutic potential of calcium phosphate-based biomaterials combined with honey in a porcine model. Osteochondral defects were surgically induced and treated with a custom-prepared composite material. Tissue regeneration was assessed using integrated macroscopic and microscopic evaluation, supported by multimodal imaging techniques. The outcomes were compared with both a spontaneous healing group and a control site with native cartilage. The composite biomaterial significantly enhanced osteochondral regeneration, with results comparable to healthy cartilage. Notably, improved structural organization and more advanced healing responses were observed in the treated group compared to spontaneous healing. The beneficial effects are attributed to the anti-inflammatory, antimicrobial, antioxidant, and immunomodulatory properties of honey, which may enhance the regenerative microenvironment and support tissue repair. These findings highlight the potential of calcium phosphate-based biomaterials combined with honey as a promising strategy for osteochondral defect treatment, improving structural, biological, and biomechanical aspects of healing.

## 1. Introduction

Osteochondral defects represent a significant clinical challenge in both human and veterinary medicine, as they involve two biologically and functionally distinct tissues—hyaline cartilage and subchondral bone. Articular cartilage plays a critical role in dissipating mechanical loads transmitted to the underlying bone, functioning as a natural shock-absorbing tissue. Its biomechanical performance is closely dependent on the structural support and integrity of the subchondral bone [1,2]. From biochemical, structural, and mechanical perspectives, cartilage and bone represent fundamentally distinct tissues [3,4]. As a result, their contrasting biological properties and markedly different regenerative potentials make the management of osteochondral defects particularly demanding [5,6].

These lesions commonly arise as a consequence of trauma, degenerative joint disease, osteochondrosis, inflammatory arthropathies, or ischemic changes, ultimately leading to progressive joint dysfunction, chronic pain, and the development of osteoarthritis [7,8]. Owing to the avascular, aneural, and hypocellular nature of articular cartilage [9,10], its intrinsic regenerative capacity is severely limited, rendering the effective treatment of osteochondral defects a persistent challenge in both experimental models and clinical practice [11,12,13,14].

Conventional treatment approaches for osteochondral defects, including microfracture, abrasion arthroplasty, mosaicplasty, and autologous chondrocyte implantation, frequently result in incomplete and short-term repair outcomes [15,16,17]. These techniques typically lead to the formation of fibrocartilage rather than native hyaline cartilage, thereby compromising the biomechanical integrity and long-term biological function of the repaired tissue [18,19,20,21]. Moreover, most conventional strategies fail to adequately restore the subchondral bone compartment, which plays a critical role in load transmission, shock absorption, and long-term joint stability [14,22,23]. As a result, there is increasing interest in regenerative medicine and tissue engineering approaches that aim to address both cartilage and subchondral bone regeneration in an integrated manner [24,25,26].

Regenerative medicine is an interdisciplinary field focused on the repair, replacement, or regeneration of damaged tissues and organs through the integration of cell biology, materials science, and clinical medicine [27]. Core strategies include the use of stem and progenitor cells, bioactive molecules, and biomaterial scaffolds, which together create a regenerative microenvironment that supports endogenous tissue repair processes [28]. Over the past two decades, the field has evolved from relatively simple cell transplantation approaches toward complex tissue-engineered constructs designed to recapitulate the native architecture, mechanical properties, and biological signaling of target tissues [29]. Despite significant advances, a major challenge in regenerative medicine remains the achievement of long-term functional integration of newly formed tissue with the host environment, including adequate vascularization, immunological tolerance, and mechanical stability [30], which is particularly critical in the regeneration of complex musculoskeletal tissues. Biomaterials play a central role in modern osteochondral repair. They aim to provide temporary or permanent scaffolds that support cell adhesion, proliferation, and differentiation while maintaining mechanical integrity during regeneration. Ideal biomaterials are biocompatible, biodegradable in a controlled manner, non-toxic, and capable of integrating with host tissue, while mimicking the physical, chemical, and biological properties of the native osteochondral complex [4].

Current regenerative strategies for osteochondral defect repair employ a broad spectrum of biomaterials tailored to the distinct biological and mechanical requirements of cartilage and subchondral bone. Natural polymers such as collagen, chitosan, and alginate, as well as synthetic polymers including polycaprolactone (PCL), polylactic acid (PLA), and poly(lactic-co-glycolic acid) (PLGA), are predominantly utilized for the cartilage compartment due to their favorable viscoelastic properties and ability to support chondrogenic cell behavior [21,31]. In contrast, calcium phosphate-based ceramics, including hydroxyapatite, α- and β-tricalcium phosphate, and biphasic calcium phosphate, are widely applied to the subchondral bone region owing to their chemical similarity to native bone mineral, osteoconductivity, and mechanical competence [32]. Among these materials, α-tricalcium phosphate (α-TCP) is particularly notable for its pronounced solubility, its ability to undergo a hydration-driven setting reaction, and its bioresorbable nature [33].

Consequently, increasing emphasis has been placed on multilayered or compositionally graded scaffolds that integrate polymeric and calcium phosphate phases, thereby recapitulating the intrinsic structural heterogeneity of osteochondral tissue from compliant cartilage to rigid subchondral bone. Such stratified designs enable spatially controlled cell differentiation, improved load transfer across the osteochondral interface, and enhanced integration with the surrounding host tissue [34].

Honey is one of the most complex natural foods, containing more than 200 substances, and its composition strongly depends on its botanical and geographical origin [35]. The main components of honey are sugars (approximately 80%), water (around 20%), and minor constituents such as proteins (enzymes), organic acids, vitamins, minerals, pigments, and phenolic compounds.

Phenolic compounds are responsible for the diverse color and flavor of honey and contribute significantly to its antioxidant and antimicrobial properties [11]. The acidic nature of honey (pH 3.2–4.5) further enhances its antibacterial activity, as most bacterial strains grow optimally at pH 6.5–7.5.

The antimicrobial effect of honey is attributed to both peroxide (H_2_O_2_) and non-peroxide components, including phenolic compounds, organic acids, and flavonoids. Additional contributing factors include low water activity (high osmolarity), high sugar content, the presence of bee defensin-1, and methylglyoxal [36].

The main functional constituent responsible for the honey’s stable non-peroxide antibacterial activity is methylglyoxal (MGO) compound. In addition, a rich profile of polyphenolic compounds (like syringic acid and methyl syringate in the case of used manuka honey) enhances the antioxidant and anti-inflammatory properties of composite which may consequently support osteogenic potential of osteoblasts. The functional ingredients of Manuka honey were described in detail in the work of Medvecky et al. 2023 [37].

From the point of view of osteogenic properties of honey, it was shown that the flavones and flavonoids in honey have good osteogenic potential, promoting the osteogenic differentiation of mesenchymal stem cells and accelerating bone fracture healing [38,39]. Moreover, the beneficial effects of honey on bone metabolism, osteoporosis, and fracture healing have been experimentally and clinically reported [40,41]. These effects are primarily attributed to its anti-inflammatory, antioxidant, and antimicrobial properties, as well as its ability to modulate immune responses and stimulate tissue regeneration. Honey has been shown to enhance osteoblast activity, promote angiogenesis, and support extracellular matrix formation, thereby facilitating bone repair and remodeling [42,43,44].

However, the application of honey in combination with calcium phosphate-based biocements for the treatment of osteochondral defects represents a novel therapeutic strategy that is currently limited to the field of experimental and preclinical research. Honey has been used in wound treatment since ancient times, and its therapeutic potential has been extensively confirmed by modern scientific research. Medical-grade honey, particularly Manuka honey, exhibits broad-spectrum antimicrobial activity, including effectiveness against antibiotic-resistant bacteria, primarily due to its high methylglyoxal content and non-peroxide antibacterial mechanisms [45]. In addition to its antimicrobial effects, honey promotes wound healing by maintaining a moist wound environment, reducing inflammation, stimulating angiogenesis, and accelerating epithelialization [46]. Both clinical and experimental studies have demonstrated the beneficial effects of honey in the treatment of acute and chronic wounds, burns, surgical defects, and infected wounds in both human and veterinary medicine [47,48].

In this study, we employed calcium phosphate-based biomaterials in combination with honey as a novel regenerative strategy for the treatment of osteochondral defects. Calcium phosphate biocement (αTCP—alpha tricalcium phosphate) was selected due to its well-documented osteoconductivity, biocompatibility, and structural similarity to the mineral phase of native bone, making them particularly suitable for the regeneration of the subchondral bone compartment [32]. Honey (Manuka honey) was incorporated as a biologically active adjunct owing to its anti-inflammatory, antimicrobial, antioxidant, and immunomodulatory properties, as well as its emerging role in promoting tissue regeneration and angiogenesis [42,49]. The combination of calcium phosphate-based scaffolds with honey was designed to synergistically support both structural regeneration of the osteochondral unit and biological modulation of the local healing environment, thereby addressing key limitations of conventional single-material approaches.

## 2. Materials and Methods

### 2.1. Preparation of Cement Pastes

The αTCP powdered phase was prepared by the solid-state synthesis from calcium carbonate (CaCO_3_, analytical grade, Sigma Aldrich, Saint Louis, MO, USA)/calcium hydrogen phosphate anhydrous (monetite, DCPA) (CaHPO_4_ (Ph.Eur., Fluka) mixture at 1320 °C for 2 h. The αTCP was dry milled in a planetary ball mill (Retsch PM100, 350 rpm, agate balls and vessel, RETSCH GmbH: Haan, Germany) for 2 h.

Manuka honey (M) (Activion^®^. medical grade, Advancis medical, Nottingham, UK) was dissolved in 1% NaH_2_PO_4_ (analytical grade, Sigma-Aldrich, Steinheim, Germany) as a liquid component of biocement. The liquid component contained 5% (*w*/*v*) honey and cement (αTCP + M25) was prepared by mixing of αTCP powdered phase with the liquid component at the powder to liquid ratio (P/L) equal 2.6.

### 2.2. Characterization of Microstructure, Setting Time and Mechanical Properties

The compressive strength (CS) of cements was measured on a universal testing machine (5 kN load cell, LR5K Plus, Lloyd Instruments Ltd., Bognor Regis, UK) at crosshead speed of 1 mm/min (mean + standard deviation, *n* = 4). The samples for measurement were prepared from the cement pastes by packing in stainless cylindrical form (6 mm D × 12 mm H), setting in 100% humidity at 37 °C for 10 min and soaking in SBF at 37 °C for 1 week. The phase composition of samples was characterized by X-ray diffraction analysis (Philips X PertPro, Malvern Panalytical B.V., Eindhoven, The Netherlands, using Cu Kα radiation, 50 mA, 2Θ range 20–40°).

The cement microstructure of fractured surfaces was observed by field emission scanning electron microscopy (JEOL FE SEM JSM-7000F, Tokyo, Japan) after coating with carbon. The final setting times of the cement pastes were evaluated using the Gilmore needle test [50].

### 2.3. Preparation of Cement Extracts and In Vitro Cytotoxicity Testing

Contact cytotoxicity of the αTCP and αTCP + M25 cements was evaluated using MC3T3-E1 Subclone 4 cells (ATCC CRL-2593, Manassas, VA, USA). Sterile cement samples (Ø 6 mm, 1 mm thickness) were placed in 48-well culture plates (TPP, Trasadingen, Switzerland), and cells in 400 μL of culture medium (EMEM supplemented with 10% FBS and 1% antibiotic solution; Sigma-Aldrich) were seeded onto each surface. All experiments were performed in triplicate. Cells cultured in complete medium without a sample served as the negative control (NC). The culture medium was refreshed three times per week. Cell proliferation and morphology were visualized 48 h, 8 days, and 15 days post-seeding using live/dead fluorescent staining (Fluorescein Diacetate, FDA/Propidium Iodide, PI). Observations were performed via inverted optical fluorescence microscopy (Leica DM IL LED) equipped with a blue filter. The mechanism relies on intracellular enzymes in viable cells converting FDA into a green fluorescent product, whereas PI permeates the damaged membranes of dead cells, staining them red.

The cytotoxicity of cement extracts (αTCP and αTCP + M25) was assessed as follows: 0.2 g/mL of cements was soaked in culture medium for 24 h at 37 C. Preosteoblasts MC3T3E1 (10^4^ cells/100 µL culture medium) were seeded in a 96-well cell culture plate (Saerstedt, Germany). After 24 h of cultivation, the culture medium was discarded and replaced with extracts. After 24 h of culture with extracts, the extracts were replaced with fresh culture medium and in vitro cytotoxicity was evaluated using a MTS proliferation test assay (Cell titer 96 aqueous one solution cell proliferation assay, Promega, Madison, WI, USA) and a UV–VIS spectrophotometer (Shimadzu, Kyoto, Japan) at 490 nm.

### 2.4. Experimental Animals

Large White pigs of both sexes obtained from the PD Agro Michalovce breeding farm (Slovak Republic) were enrolled in this study and randomly assigned to two experimental groups: an implant-treated group (*n* = 5) and an spontaneous healing process group (*n* = 5). Both groups were used to assess the treatment of the osteochondral defect (Figure 2), and the newly formed tissue was subsequently compared with healthy hyaline cartilage. The animals had a mean body weight of 97.9 ± 4.89 kg (mean ± SD).

Pigs were selected as the experimental model due to their comparable joint size, adequate cartilage thickness, favorable handling characteristics, and widespread availability, making them well suited for osteochondral research. Throughout the in vivo experiment, the animals were housed at the Swine Clinic of the University of Veterinary Medicine and Pharmacy in Košice (Slovak Republic) and were monitored daily by trained animal care personnel. All pigs received a standardized diet and had ad libitum access to drinking water for the duration of this study. The experimental protocol was approved by the State Veterinary and Food Administration of the Slovak Republic (approval No. 4650/17-221).

#### 2.4.1. Anesthesia and Perioperative Management

Premedication of the animals was achieved by intramuscular administration of a solution prepared by mixing zolazepam (50 mg/mL; Zoletil^®^ 100 Vet., Virbac, Nice, France), ketamine (2.5 mL; Ketamidor^®^ 100 mg/mL, VetViva Richter GmbH, Wels, Austria), xylazine (2.5 mL; Xylased^®^ 100 mg/mL, Bioveta SK spol. s r.o., Nitra, Slovakia), and butorphanol (1.5 mL/100 kg body weight; Butomidor^®^ 10 mg/mL, VetViva Richter GmbH, Wels, Austria). Subsequently, a cannula was placed in the left lateral auricular vein (*v. auricularis lateralis sinistra*) and used for intravenous administration of a solution consisting of zolazepam, ketamine, and xylazine at a dose of 0.5–1.0 mL to induce general anesthesia.

#### 2.4.2. Unilateral Surgical Formation and Treatment of Osteochondral Defects

A midline skin incision was made over the left stifle joint, extending distally from the medial patellar ligament (*ligamentum patellae mediale*) to the tibial tuberosity (*tuberositas tibiae*). A standardized osteochondral defect measuring 10 mm in diameter and 10 mm in depth was subsequently created in the load-bearing region of the medial femoral condyle (*condylus medialis*). The defect was produced using a commercially available osteochondral autograft transfer system (OATS; Arthrex, Naples, FL, USA), allowing for precise control of both the defect diameter and depth.

To stimulate regeneration via bone marrow activation, the subchondral bone was perforated to a predefined depth, enabling bleeding from the marrow cavity. In the first experimental group (*n* = 5), the cartilage and subchondral bone defects were filled with sterile αTCP + M25 biocement paste. Conversely, in the second experimental group (*n* = 5), an osteochondral defect of the same size was left to heal spontaneously, without the application of any therapeutic procedure or biomaterial (Figure 2).

Both experimental groups (αTCP + M25 biocement-treated defects and untreated osteochondral defect) were evaluated in comparison with a control site represented by the lateral femoral condyle (*condyles lateralis*) of the same left stifle joint, which retained native cartilage and subchondral bone. After defect treatment, the muscle layers and skin were closed in a layered manner using resorbable sutures, and the surgical wound was protected with a liquid aluminum bandage. No external fixation or immobilization techniques, such as splints or casts, were used in any of the animals.

**Figure 1:**
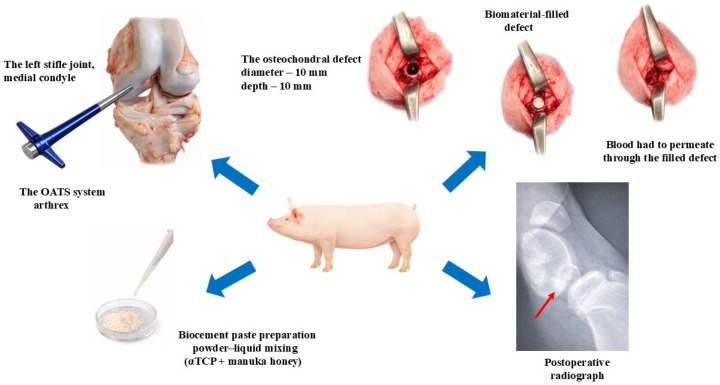
Schematic representation of the osteochondral defect study design. Intraoperative image showing a unilateral defect created in the medial condyle of the porcine knee joint. The defect was filled with αTCP + M25 biocement paste (*n* = 5), whereas in the second group the defect was left untreated to allow for spontaneous healing (*n* = 5). A control X-ray examination was performed to confirm proper filling of the osteochondral defect.

Conventional lateral-view radiographs of the porcine knee joint were obtained to verify the creation of the osteochondral defect and its complete filling with biocement paste in the first experimental group (Figure 3). Following surgery, the animals were housed individually in solid-floor pens with straw bedding. Immediate full weight bearing was permitted, and the pigs were allowed to return to normal, unrestricted activity. A schematic illustration of the osteochondral defect study design is presented in Figure 1.

**Figure 2:**
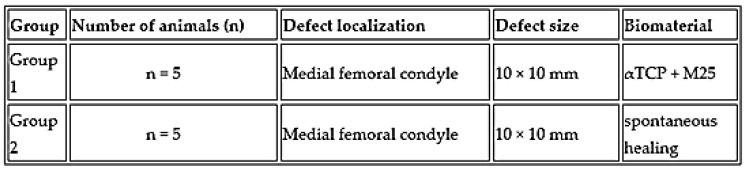
Experimental design of animals and biomaterials used in our study.

**Figure 3:**
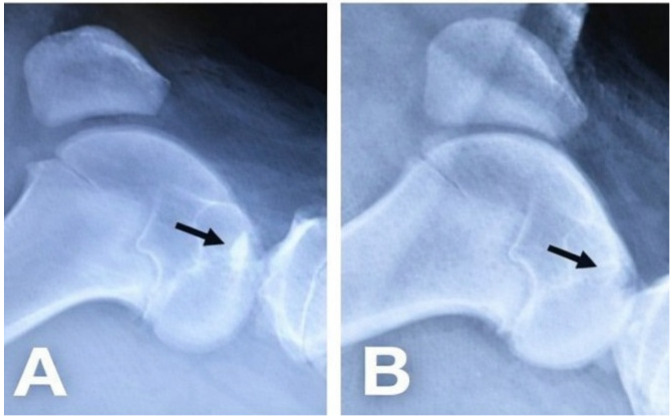
Control X-ray image after surgery in the knee joint, which we used to verify the correctness of the created defects and the implantation of biocement. (**A**). defect filled with biocement αTCP + M25, (**B**). created defect left to heal spontaneously.

#### 2.4.3. Postoperative Management

Postoperative management comprised prophylactic antimicrobial treatment delivered via repeated intramuscular injections of the broad-spectrum antibiotic oxytetracycline dihydrate (1 mL/10 kg body weight; Alamycin LA a.u.v., Norbrook, Newry, UK), administered every other day for a total of seven days. Analgesic and anti-inflammatory therapy was provided by intramuscular administration of the non-steroidal anti-inflammatory drug flunixin meglumine (2.2 mg/kg body weight; Flunixin a.u.v., Norbrook, Newry, UK), given once daily for seven consecutive days. Animals were closely monitored and clinically examined throughout the entire postoperative follow-up period until the end of the experiment. Clinical evaluation included assessment of general health status, body temperature, fecal consistency, degree of limb pain, and the condition of the surgical site. Wound healing was evaluated for signs of inflammation, including erythema, swelling, increased local temperature, discharge, and pain upon palpation. In addition, lameness was systematically scored to document functional outcome. Regular clinical assessments ensured continuous monitoring of animal welfare, pain control, and locomotor performance.

#### 2.4.4. Animal Euthanasia and Tissue Collection

Animals were humanely euthanized six months after the implantation procedure. Prior to euthanasia, sedation was induced by intramuscular administration of azaperone at a dose of 2 mg/kg body weight (Stresnil 40 mg/mL, Janssen Pharmaceutica, Beerse, Belgium). Euthanasia was subsequently performed by intravenous administration of thiopental at a dose of 90 mg/kg body weight (Thiopental VUAB 1.0 g, VUAB Pharma a.s., Roztoky, Czech Republic).

After euthanasia, the muscle tissue of the left pelvic limb was carefully removed, and the knee joint capsule was opened to permit gross macroscopic evaluation of the articular cartilage surface. The cartilage was visually examined, photographically documented, and harvested for further analyses. The osteochondral defect sites were then subjected to macroscopic assessment, as well as histological, immunohistochemical, and radiographic evaluation. The medial femoral condyle of the contralateral right knee joint served as a control.

### 2.5. Evaluation of Tissue Regeneration

#### 2.5.1. Gross Macroscopic Evaluation

After removal of the surrounding soft tissues, the osteochondral defect area and adjacent structures were subjected to gross macroscopic assessment. The evaluation focused on the surface characteristics of the newly formed cartilage, including surface smoothness, color, opacity, translucency, tissue consistency, degree of defect filling, and integration at the defect margins with the neighboring native cartilage. These features were compared with those of the intact, physiologically normal surrounding cartilage tissue.

In addition, the articular surfaces of the distal femoral epiphysis and proximal tibial epiphysis, as well as the synovial membrane and knee joint capsule, were carefully inspected for signs of inflammation or pathological changes, such as osteophyte formation. The repair tissue formed within the osteochondral defects in both experimental groups (biocement-treated defects and spontaneous healing process) was semiquantitatively evaluated using the International Cartilage Repair Society (ICRS) scoring system for cartilage repair, as previously described in [51] (Figure 5).

#### 2.5.2. Histological and Immunohistochemical Evaluation

Tissue samples encompassing the regenerated osteochondral defect region were collected as bone–cartilage cylinders measuring 10 mm in diameter and 10 mm in length, harvested using an osteochondral autograft transfer system (OATS; Arthrex, Naples, FL, USA).

Specimens were fixed in 10% neutral buffered formalin at room temperature. Following fixation, samples were decalcified in 25% EDTA medium (CentralChem, Slovakia). Decalcification endpoint was verified by mechanical testing (needle/forceps). After decalcification, tissues were processed through graded series of ethanol, cleared in xylene and embedded in paraffin.

##### H-E Staining Methods

This combination of dyes enables clear visualization of cellular and extracellular structural details. Tissue sections were deparaffinized in xylene and rehydrated through graded series of ethanol. Sections were subsequently stained with hematoxylin for 5 min, rinsed, and counterstained with eosin for 3 min. After staining, slides were dehydrated in graded alcohols, cleared in xylene, and mounted using a synthetic resin medium (Entellan; Merck, Germany).

##### Safranin-O/Fast Green Staining Methods

Safranin-O staining (red to orange) reflects the presence of a matrix rich in sulfated glycosaminoglycans, that are found in hyaline cartilage and chondroid tissue. In contrast, Fast Green serves as a counterstain for collagen-rich extracellular matrix and bone, producing a green to blue-green coloration depending on the specific formulation and degree of differentiation.

Tissue sections were deparaffinized in xylene and rehydrated through graded series of ethanol. Nuclei were stained with Weigert’s iron hematoxylin for 5 min, followed by rinsing in running water. The sections were subsequently rinsed in acid alcohol, followed by three washes in distilled water. Sections were stained in 0.02% Fast Green for 1.5 min and rinsed briefly in 1% acetic acid for 30s to remove excessive Fast Green and sharpen the contrast.

Safranin O staining. Slides were stained with 0.1% Safranin O for 15 min, dehydrated rapidly through graded series of ethanol, cleared in xylene, and cover slipped with a mounting medium.

##### Immunohistochemical Analysis

Histological sections of decalcified tissues were deparaffinized and rehydrated. Endogenous peroxidase activity was blocked with 3% H_2_O_2_ with methanol. Before incubating the primary antibody, antigen retrieval was performed using PT-Linker (Dako) and High pH Target Retrieval Buffer. For immunohistochemical detection, the all sections were treated with primary antibodies. Primary antibodies were labeled using a two-stage indirect immunoperoxidase technique. Primary and secondary antibodies were applied at the appropriate titer (Figure 6). Positive cells for colorimetric immunohistochemical analyses were visualized by diaminobenzidine (DAB; SIGMA-ALDRICH, Co). The nuclei were counterstained using Mayer’s hematoxylin (32750; SIGMA-ALDRICH, Co; St. Louis, MO, USA) and cover-slipped with Pertex (Histolab Products AB; Göteborg, Sweden).

##### Antibody Specificity

In order to establish the specificity of the immunohistochemistry, a negative control test was carried out. Primary antibodies were omitted in all immunohistochemical methods for negative control test. The negative control test was conducted in all groups.

Tissue sections were examined in high-power fields (magnification ×400) and the pictures were taken using an Olympus BX50 light microscope with an Olympus SP350 camera.

**Figure 4:**
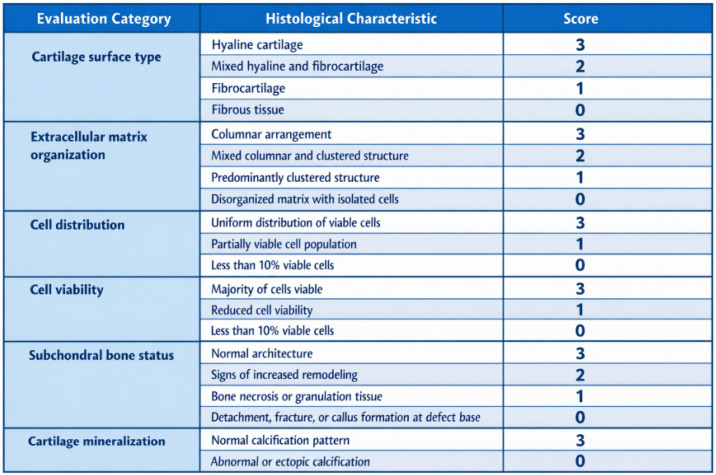
Modified ICRS visual histological scoring system for evaluation of cartilage repair [52].

The structural quality of the repair cartilage was assessed using the ICRS Visual Histological Assessment Scale, based on criteria including cartilage surface characteristics, extracellular matrix composition, cellular distribution and viability, integrity of the subchondral bone, and cartilage mineralization, as previously described in [52] (Figure 4).

**Figure 5:**
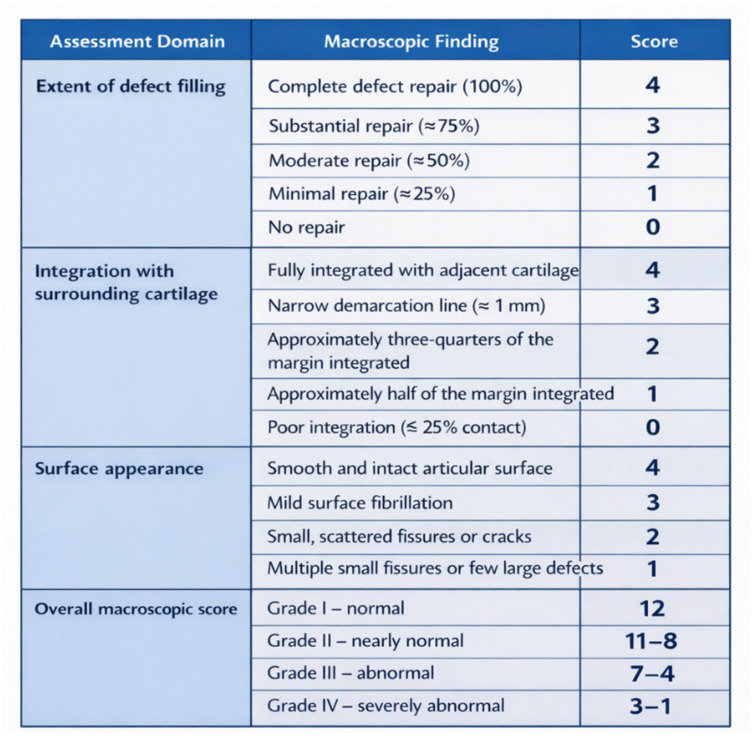
Modified ICRS macroscopic scoring system for evaluation of cartilage repair [51].

**Figure 6:**
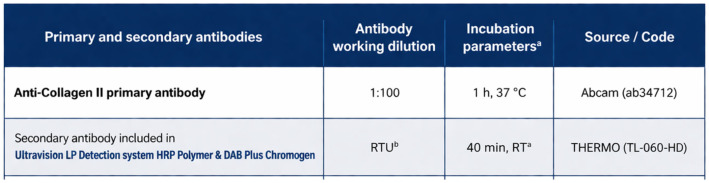
Primary and secondary antibodies used in our study.

#### 2.5.3. Biochemical and Metabolomic Analysis of Blood Serum

##### Clinical-Biochemical Analysis

The determination of clinical-biochemical parameters in a set of blood serum samples was carried out using the Skyla HB1 biochemical analyzer (Skyla Corporation, Hsinchu, Taiwan) and two types of diagnostic discs. The first was a general biochemical panel comprising 15 parameters: ALB (albumin), ALP (*alkaline phosphatase*), ALT (*alanine aminotransferase*), AST (*aspartate aminotransferase*), BUN (blood urea nitrogen), CREA (serum creatinine), GGT (*γ-glutamyl transferase*), GLU (glucose), TBIL (total bilirubin), TC (total cholesterol), TP (total proteins), UA (uric acid), GLOB (globulin), UREA (urea), and the A/G ratio (albumin/globulin). The second group consisted of a metabolic panel with 16 indicators: ALB, ALT, AST, BUN, Ca (calcium), Cl (chlorides), CREA, GLU, K (potassium), Na (sodium), PHOS (phosphates), TP, UA, the A/G ratio, GLOB, and UREA. For the analysis, 200 µL of blood serum was applied to the respective disc, which was stored in a refrigerator at a temperature of 2–8 °C until processing. The analysis of a single sample in the device lasted 15 min, as preset by the software.

##### UHPLC Analysis

Selected biomarkers (creatinine, tyrosine, tryptophan, methionine, hydroxyproline, adenosine, hypoxanthine, and neopterin) were determined using a Dionex UltiMate 3000 RS UHPLC system (Thermo Fisher Scientific, Waltham, MA, USA). The separation of target compounds was achieved on a YMC-Triart PFP chromatographic column (150 × 3.0 mm I.D., particle size 1.9 μm, pore size 12 nm; YMC Europe GmbH, Dinslaken, Germany). During the analysis, the column temperature was kept constant at 30 °C (±0.5 °C), and the total run time was 20 min. A sample injection volume of 10 μL was used for both standards and serum samples. The mobile phase consisted of methanol (component A) and deionized water containing 0.1% formic acid (component B). Gradient elution was applied, with the proportion of component B adjusted as follows: 100–70% (0–15 min), 70–100% (15–15.1 min), and 100% (15.1–20 min). The flow rate was maintained at 0.5 mL/min. Detection of analytes was performed using a diode array detector (DAD; wavelengths 200 and 220 nm) coupled in series with a fluorescence detector (FLD; excitation at 280 nm and emission at 350 nm). The obtained chromatographic data were processed and analyzed using Chromeleon 7.2 Chromatography Data System software.

#### 2.5.4. Radiological Evaluation

Six months after the surgical procedure, post-mortem imaging examinations were performed to assess the regenerative response of the articular cartilage and subchondral bone in the left porcine knee joints. The evaluation comprised conventional radiographic imaging (X-ray) and magnetic resonance imaging (MRI).

Radiographic assessment was carried out using lateral-view projections of the knee joint acquired with a digital X-ray system (Philips Digital Diagnost, Delft, The Netherlands). Magnetic resonance imaging (MRI) was performed in two acquisition planes using a specialized Zenith solenoid knee coil, allowing for optimal visualization of the articular cartilage. The sagittal plane was acquired using a proton density fat-suppressed (PD-FS) sequence. Imaging parameters included a repetition time (TR) of 3805 ms and an echo time (TE) of 36 ms.

The second acquisition plane was axial, using a T2-weighted sequence (T2 AX), with imaging parameters of a repetition time (TR) of 4500 ms and an echo time (TE) of 84 ms.

Images were obtained with ultra-high spatial resolution and a slice thickness of 3.5 mm using a 1.2 T open MRI system (Hitachi Oasis, Hitachi Medical Systems Holding AG, Tokyo, Japan).

All MR images were digitally transferred to the TOMO CON PACS network and subsequently processed and evaluated using dedicated Tatra Med image analysis software Začiatok formulára.

## 3. Results

### 3.1. XRD, FTIR Analysis of Cements

**Figure 7:**
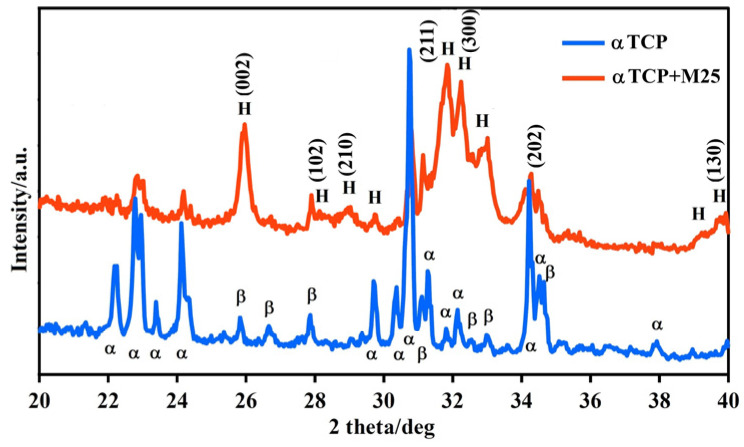
XRD patterns diffraction analysis of αTCP and αTCP + M25.

XRD diffraction analysis of starting powdered cement phase showed the formation of major αTCP phase (JCPDS 29-0359) with about 9% content of βTCP secondary phase (JCPDS 09-0169) (Figure 7). More than 80% conversion of αTCP to nanocrystalline hydroxyapatite (JCPDS 72-1243) resulted from comparison with αTCP + M25 patterns of composite biocements after 7 days soaking in SBF at physiological conditions and remains of βTCP phase can be also visible in patterns.

**Figure 8:**
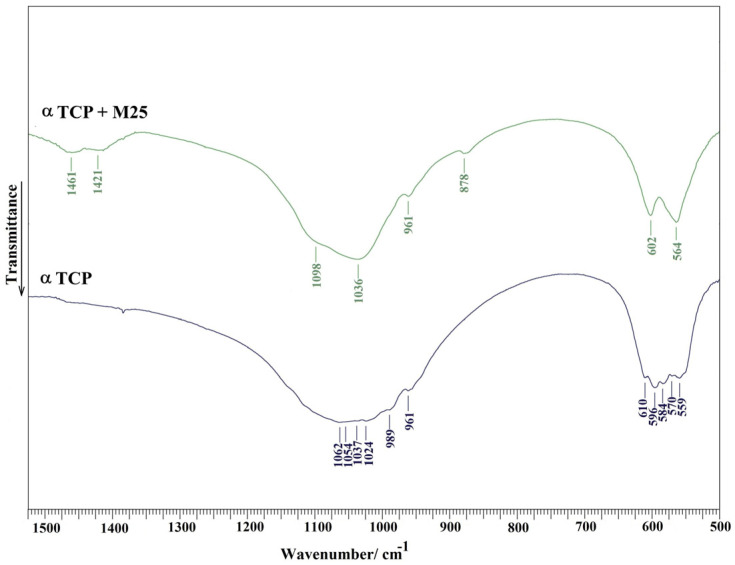
FTIR spectra of origin αTCP phase and αTCP + M25 composite cement after 7 days soaking in SBF and 37 °C.

The spectrum revealed a significant attenuation of α-TCP bands, which originate from phosphate group stretching (1062–961 cm^−1^) and bending (610–559 cm^−1^) vibrations [53]. Concurrently, bands characteristic of a nanocrystalline hydroxyapatite (HAP) phase were clearly identified. These include phosphate stretching modes at 1098, 1032, and 961 cm^−1^ (antisymmetric *υ_3_* and symmetric υ_1_ stretching) and bending modes at 602 and 564 cm^−1^ (υ_4_ bending) [54]. The nanocrystalline character of the HAP is evidenced by the absence of hydroxyl group vibrations at 3560 and 630 cm^−1^ (stretching and vibrational modes) [55]. Furthermore, the resulting HAP phase incorporates minor carbonate substitution at B-sites, indicated by bands at 1460 and 1420 cm^−1^ (asymmetric υ_3_ stretching) and 875 cm^−1^ (υ_2_ bending) (Figure 8) [56].

### 3.2. Characterization of Biocement Microstructure, Setting Time and Compressive Strength

**Figure 9:**
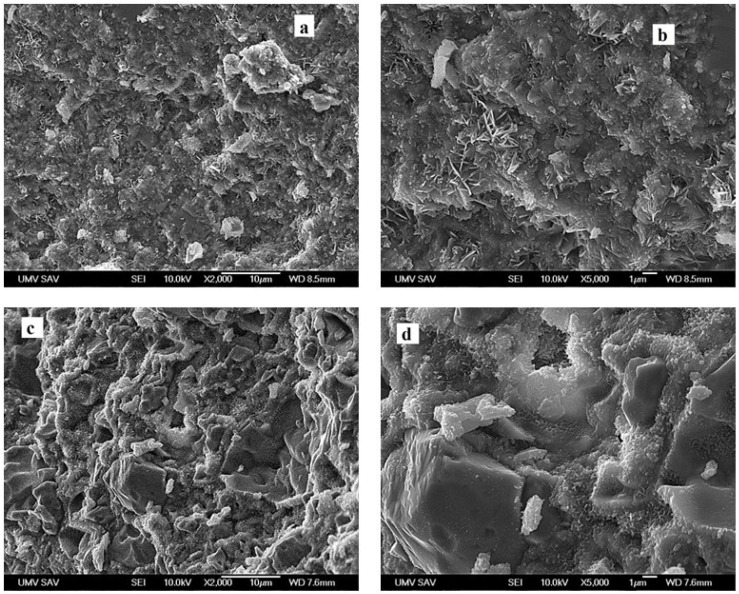
Microstructures of biocement (**a**,**b**)—αTCP, (**c**,**d**)—αTCP + M25. (**a**) The mixture of irregularly shape and granular agglomerates of HAP particles were identified in the microstructure of αTCP biocement. (**b**) The rod- and plate-like thin HAP nanoparticles (length up to 1 μm) and represent the main morphological type of αTCP biocement. (**c**) The addition of manuka honey in αTCP + M25 samples supported the formation of dense microstructure with a higher fraction of micropores around 1 μm in size. (**d**) Compact irregularly shaped agglomerates (up to 5 μm size), which can represent the remains of origin αTCP particles surrounded by fine HAP particles, were found in microstructure of fractured sample.

The mixture of irregularly shape and granular agglomerates of HAP particles were identified in the microstructure of αTCP biocement, which are reason for presence of the spherical and irregularly shaped macropores with size up to 10 μm due to pulling out of weakly bonded agglomerates of HAP nanoparticles from cement matrix (Figure 9a). In addition, agglomerates are composed of the rod- and plate-like thin HAP nanoparticles (length up to 1 μm) and represent the main morphological type of objects (Figure 9b), which are mutually interconnected with very fine nanocrystalline granularly shaped HAP particles (submicrometric size) in cement matrix. The addition of manuka honey in αTCP + M25 samples supported the formation of dense microstructure with a higher fraction of micropores around 1 μm in size located between agglomerates of very fine hydroxyapatite matrix. In composite cement (αTCP + M25) is visible dense microstructure in relation to compactness of agglomerates. The apparent density of the hardened cements was independent following the addition of honey to the cement and was close to 51 ± 2% of theoretical HAP density (Figure 9c). Compact irregularly shaped agglomerates (up to 5 μm size), which can represent the remains of origin αTCP particles surrounded by fine HAP particles, were found in microstructure of fractured sample (Figure 9d).

The final setting times of αTCP + M25 sample prolonged almost twice as compared with αTCP cement and achieved 28 min. Moreover, compressive strength of αTCP (39 MPa) was significantly higher than αTCP + M25 (27 MPa).

### 3.3. Cytotoxicity Analysis of Cement Extracts and Osteoblast Proliferation on Cement Surfaces

**Figure 10:**
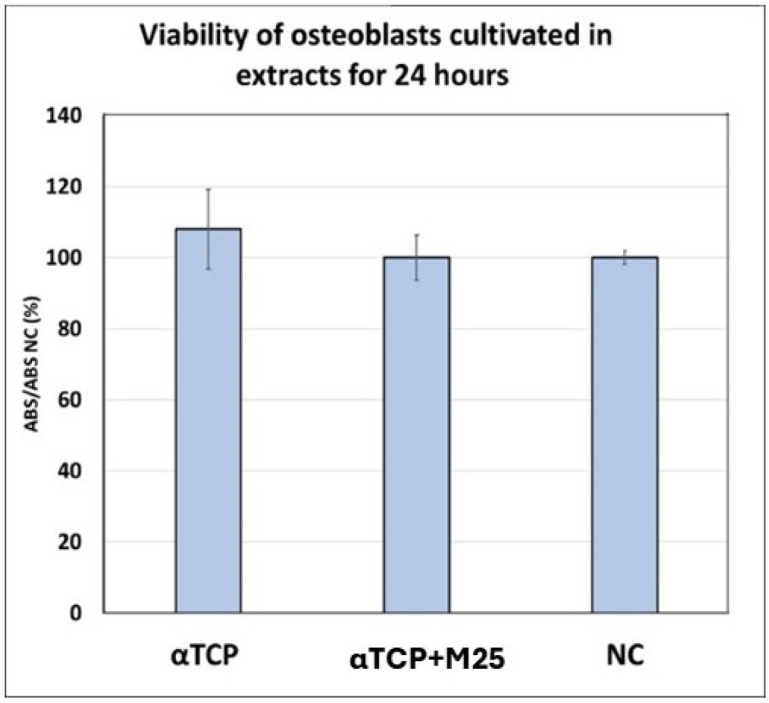
Viability of osteoblasts cultivated in extracts of tested biocements.

**Figure 11:**
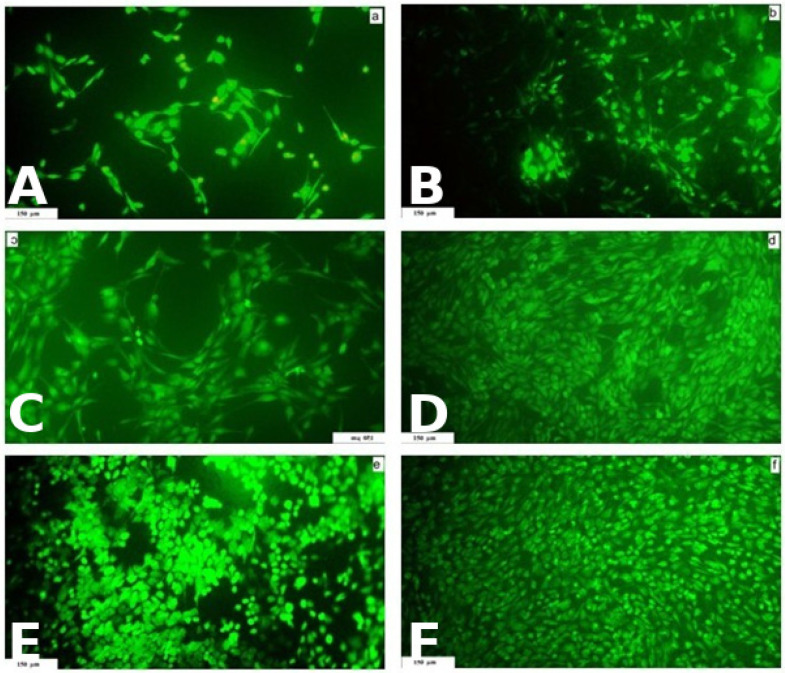
Live/dead staining of cells growing on cement surfaces. αTCP: (**A**) 48h; (**C**) 8 days; (**E**) 15 days. αTCP + M25**:** (**B**) 48h; (**D**) 8 days; (**F**) 15 day.

No cytotoxicity was observed in the cement extracts according to the standard MTS assay. The viability of osteoblasts cultured in αTCP and αTCP + M25 extracts reached 108 ± 11% and 100 ± 10% of the negative control, respectively. These values are significantly higher (*p* < 0.05) than the 70% threshold typically used to define cytotoxicity of extracts (Figure 10). Furthermore, live/dead staining confirmed the non-cytotoxic nature of the cements after 48 h (Figure 11A,B), 8 days (Figure 11C,D), and 15 days of culture (Figure 11E,F). The cells exhibited excellent adherence, spreading, and population density across all samples. Notably, no dead cells were detected on the sample surfaces, regardless of cement composition. Cell density increased over time, with the αTCP + M25 cement showing a more confluent population than the αTCP (without honey) after 8 and 15 days of cultivation.

### 3.4. Gross Macroscopic Evaluation

Based on the preliminary macroscopic evaluation, we concluded that the application of the selected biomaterial significantly influenced the healing process of the osteochondral defect.

During the postoperative observation period, the basic vital parameters of all animals were regularly monitored. In the first few days following surgery, mild inflammatory signs were observed in two pigs from the experimental group in which spontaneous healing of the induced defect was evaluated. A comparable condition was also noted in one animal treated with biocement. In all affected cases, the inflammatory symptoms and mild lameness resolved within several days. All animals received standardized postoperative injectable therapy consisting of antibiotics and non-steroidal anti-inflammatory drugs (NSAIDs), which likely contributed to the resolution of these clinical signs.

The surgical wounds remained clean and dry throughout the recovery period, with the exception of the three cases mentioned above, which did not show long-term persistent signs of inflammation. The functional status of all experimental animals remained within physiological limits, with no observed deviations from normal locomotor activity. Following the removal of soft tissues and the opening of the examined knee joints, the articular surfaces, synovial membranes, and adjacent periarticular tissues were intact, without macroscopic evidence of abrasion or inflammatory changes.

**Figure 12:**
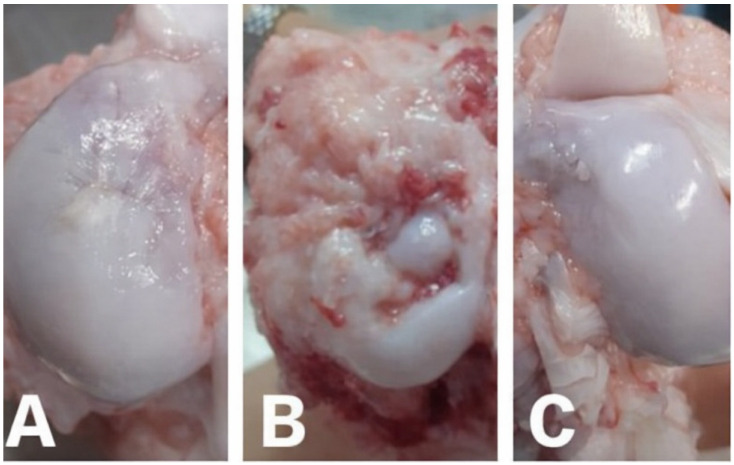
Macroscopic evaluation of the healed knee joint in the αTCP + M25 biocement group, the spontaneous healing group, and the control group 6 months after surgery. (**A**) In the honey cement group, we observed successful tissue regeneration in the previously created osteochondral defect. (**B**) The spontaneous healing group showed soft tissue hypertrophy and the presence of fibrous tissue. (**C**) For comparison, we include the appearance of healthy, articular hyaline cartilage.

**Figure 17:**
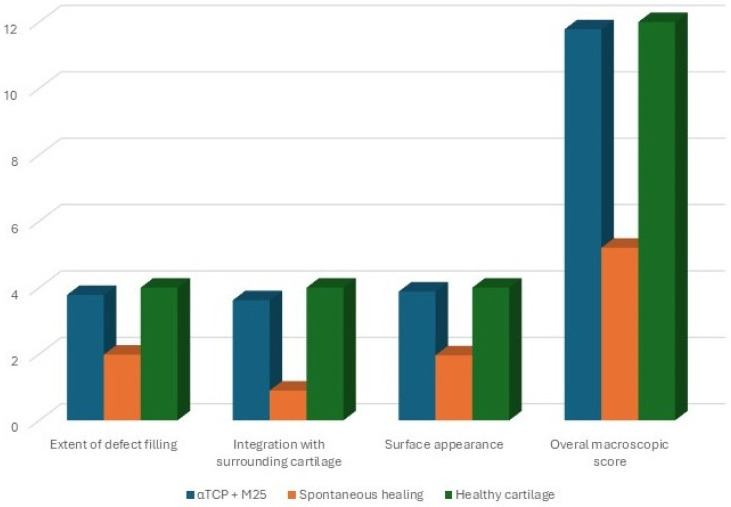
Visual macroscopic assessment by ICRS provided a quantitative comparison of the repaired cartilage tissue in both experimental groups (αTCP + M25 biocement group and spontaneous healing group) compared to healthy, hyaline cartilage [51].

Macroscopically, the created defect remained identifiable in both experimental groups. The healing process and the degree of defect closure varied during the recovery period, as reflected by differences in tissue appearance and the extent of newly formed tissue among individual animals. Six months after implantation of the honey-based biocement, the treated defects demonstrated the most advanced cartilage regeneration, characterized by the presence of shiny, smooth, and whitish newly formed tissue. The newly formed tissue was well integrated with the adjacent native cartilage. Neocartilage formation appeared to initiate at the margins of the defect and progressively extended toward its center. The healing response led to a consistent filling of the defect with tissue exhibiting a relatively smooth, uniform, and glossy surface, closely resembling the adjacent healthy articular cartilage (Figure 12C). The surface level of the neocartilage was aligned with that of the surrounding cartilage (Figure 12A). In animals exhibiting spontaneous healing, a distinct demarcation between the native and regenerated tissue was observed at the end of the recovery period. In this group, repair of the irregular cartilage surface was evident, with an estimated defect coverage of approximately 50% or less. Several specimens demonstrated fissures in the central region of the defect or localized depressions within the repair tissue. In some cases, soft tissue ingrowth originating from the site of the original defect was also noted (Figure 12B). According to the criteria of the International Cartilage Repair Society (ICRS), the biocement-treated group exhibited normal repair (Grade I; score 12), whereas the spontaneous healing group showed an abnormal repair response (Grade III; score 6) (Figure 17).

### 3.5. Histological and Immunohistochemical Evaluation

**Figure 13:**
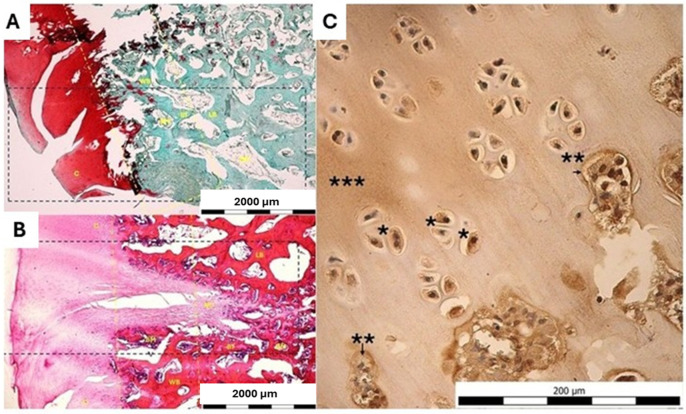
Histological evaluation of osteochondral defect after biocement therapy. (**A**) The articular cartilage (C) exhibits strong Safranin O positivity, indicating preserved proteoglycan-rich matrix. The defect region (black dashed line) extends from the cartilage into the subchondral compartment (yellow dashed line). The underlying bone is composed of trabecular bone (BT) with areas of woven bone (WB) and more mature lamellar bone (LB). The transition between cartilage and bone is irregular, reflecting ongoing osteochondral remodeling. (**B**) The corresponding H&E section demonstrates the structural organization of the defect area (black dashed line), with cartilage (C) transitioning into regions of newly formed cartilage (NC) and underlying bone trabeculae (BT). Subchondral bone (yellow dashed line) exhibits the both woven bone (WB) and lamellar bone (LB) trabeculae. Intertrabecular spaces are occupied by bone marrow (BM). The overall morphology indicates active tissue remodeling within the osteochondral unit. (**C**) Immunohistochemical staining for type II collagen was used to evaluate the presence of hyaline cartilage-specific extracellular matrix and to assess the quality of cartilage repair tissue. Immunohistochemical staining for collagen type II demonstrated strong positivity within the cytoplasm of chondrocytes (*), as well as in both the territorial (**) and interterritorial (***) extracellular matrix, consistent with a hyaline cartilage formation following the injury.

In the first group of animals treated with biocement, histological evaluation revealed a macroscopically smooth cartilage surface. Microscopically, a thin layer of flattened cells of mesenchymal origin was identified at the periphery of the cartilage surface, overlying the cartilage tissue. Collagen fibers were locally present within the cartilage matrix. Within the defect area, a fibrocartilaginous component was observed, with chondrocytes arranged in rows, gradually transitioning into hyaline cartilage characterized by chondrocytes located within lacunae. In some areas, chondrocytes formed isogenous groups. A typical highly vascularized fibrotic callus was not observed in the evaluated specimen, which may indicate an advanced stage of cartilage repair. No inflammatory elements or leukocyte infiltration were detected within the cartilage tissue. This result was also confirmed by immunohistochemical staining for collagen type II, which demonstrated strong positivity within the cyto-plasm of chondrocytes, as well as in extracellular matrix, consistent with a hyaline cartilage formation following the injury (Figure 13C).

Overall, the histomorphological appearance of the cartilage was favorable, with a clear predominance of cartilage tissue over fibrous components. The interface between fibrocartilage and hyaline cartilage was not sharply demarcated, suggesting ongoing or successfully completed remodeling and regeneration of the cartilage tissue (Figure 13A,B).

Histological evaluation of animals with spontaneous healing revealed predominant filling of the defect area with a well-developed fibrotic callus composed of densely packed, irregularly arranged collagen fiber bundles. The tissue exhibits a high degree of cellularity, with elongated spindle-shaped cells consistent with a mesenchymal/fibroblastic phenotype. No cartilaginous islands or hyaline cartilage-like structures are observed within the repair tissue, that indicates the absence of chondrogenic differentiation. The extracellular matrix is predominantly fibrous, with no features of typical cartilage formation. The fibrotic tissue is highly vascularized, with numerous blood vessels distributed throughout the callus, supporting active tissue remodeling and granulation-like characteristics.

**Figure 14:**
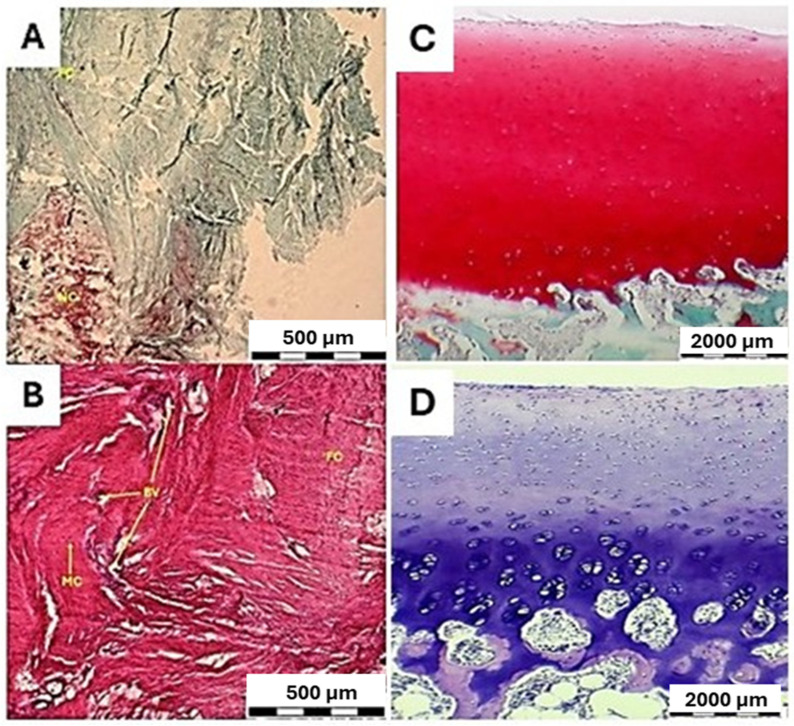
Histological appearance of articular cartilage repair by spontaneous healing (**A**,**B**) and histological appearance of healthy hyaline cartilage (**C**,**D**). (**A**) Safranin O staining shows the defect area predominantly filled with a fibrotic callus (FC), composed of collagen-rich matrix with minimal proteoglycan-associated staining. Areas of new cartilage (NC) are limited. The tissue contains blood vessels (BV) and population of mesenchymal-like cells. (**B**) The corresponding H&E section demonstrates a dense fibrotic callus (FC) with abundant fibrous extracellular matrix, populated by mesenchymal-type cells (MC) and vascular structures (BV). With H&E staining methods no hyaline cartilage or cartilage-like tissue is present within the fibrotic region. (**C**) Safranin O staining demonstrates intact articular cartilage with intense and homogeneous red staining, indicating a sulfated glycosaminoglycan-rich extracellular matrix characteristic of healthy hyaline cartilage. The cartilage surface is smooth and continuous, with preserved structural organization. (**D**) The corresponding H&E section confirms the normal morphology of articular cartilage with chondrocytes distributed within lacunae and no evidence of structural disruption or degeneration.

Histomorphological appearance corresponds to fibrous repair tissue, without evidence of cartilage regeneration (Figure 14A,B).

**Figure 18:**
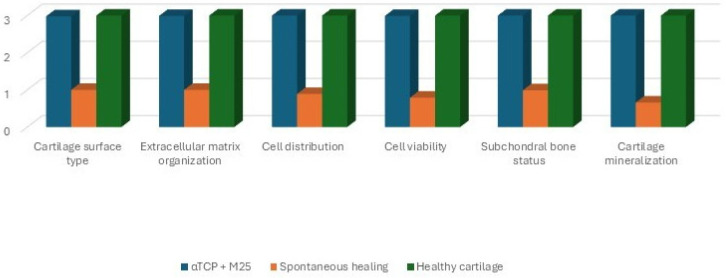
Visual histological evaluation by ICRS allowed for quantitative comparison of the repair cartilage tissue between the two experimental groups (αTCP + M25 biocement group and spontaneous healing group) [52].

Both groups of animals were compared with healthy hyaline cartilage, which exhibits a smooth surface, shows a typical organization without histomorphological changes or structural defects, and contains chondrocytes arranged within lacunae (Figure 14C,D). The ICRS Visual Histological Assessment enabled a quantitative comparison of cartilage repair tissue between the biomaterial-treated group, the artificial cyst group, and healthy cartilage (Figure 18).

### 3.6. Biochemical and Metabolomic Analysis of Blood Serum

As part of the cartilage regeneration process using biomaterial, a clinical-chemical evaluation was performed to monitor selected biochemical parameters and targeted metabolites of blood serum at five time points (I—the day before surgery, II—the day of surgery, III—one month, IV—two months, and V—three months after surgery).

In relation to protein synthesis and amino acid metabolism, ALB, GLOB, TP, and the A/G ratio were monitored. The results from experimental samples indicated increased values of GLOB and TP in stages III–V. Elevated TP and GLOB levels (hyperproteinemia) can be explained by increased collagen production, activation of the immune system, and the wound healing process. Conversely, a decreased A/G ratio indicates the presence of inflammation, which may slow wound healing. Sufficient protein availability is also supported by slightly elevated BUN levels in stages III and IV. In stage V, a gradual return toward physiological values was observed.

Additional indicators of the connective tissue regeneration process include enzyme activities of ALT, AST, GGT, and ALP. In some samples, significant changes in ALT and AST activities were observed during stages III and IV, approaching physiological norms in the final monitoring stage, confirming the production of necessary proteins and growth factors. In the case of ALP, decreased activity was observed in stages II–V compared to the reference range. Excessive oxidative stress or inflammation may suppress ALP gene expression, thereby slowing extracellular matrix mineralization. Reduced ALP levels may also be associated with decreased PHOS levels, which began to increase only after 3 months post-surgery. GGT remained within normal levels throughout the observation period, with minor fluctuations. GLU and TC levels remained relatively stable during the transplantation process, which is crucial in this case, as high glucose levels could slow collagen formation and worsen scar quality. Conversely, very low cholesterol levels could reduce the synthesis of hormones necessary for tissue remodeling. Mineral levels, except for PHOS, remained within physiological ranges throughout the monitoring period.

Another objective of this study was to monitor the concentration levels of targeted metabolites in blood serum samples over the same time intervals and to evaluate the relationship between quantitative analysis and the regeneration process. A key amino acid of collagen is hydroxyproline, a direct marker of healing and integration of transplanted connective tissue. Slightly elevated concentrations were observed on the day of surgery, with a significant increase in stages III and IV, when intensive tissue remodeling is expected. A decrease below reference intervals was observed in creatinine, tyrosine, tryptophan, and adenosine during stages II–IV.

Creatinine, an indirect marker, primarily reflects changes in tissue metabolism and catabolism. Low amino acid levels may indicate reduced immune activity, affecting graft tolerance and organism adaptation. Adenosine, as part of purine metabolism, acts as an anti-inflammatory marker and signals transplant tolerance. Low concentrations were also observed for neopterin in stage II, while slightly higher levels compared to other values were recorded in stages III and IV. Low levels may result from reduced immune activation, which gradually increased.

Neopterin, one of the most sensitive markers of immune response, showed a significant increase in stages III and IV, indicating elevated immune system activity, often associated with transplant rejection.

Methionine and hypoxanthine levels fluctuated within reference ranges during the regeneration process, with minimal deviations. The most significant deviations in methionine concentration were observed in stage II. However, a positive finding is the decrease in its levels in the final stage.

**Figure 15:**
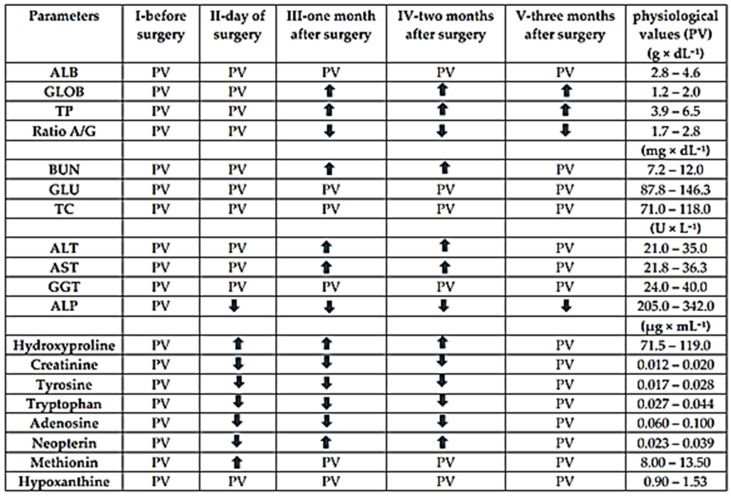
Biochemical and metabolomic blood serum analysis results.

The process of connective tissue regeneration requires a longer period for the stabilization of physiological processes after the procedure, as evidenced by the normalization of both biochemical and metabolomic parameters in the final stage. The changes in individual blood serum parameters over time are shown in Figure 15.

### 3.7. Radiological Evaluation

**Figure 16:**
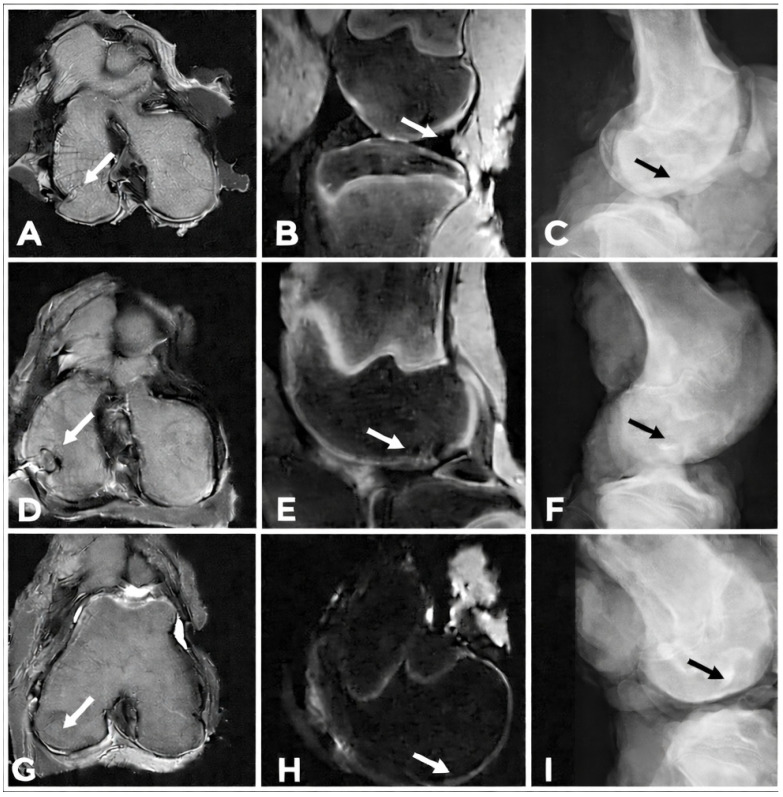
(**A**,**B**,**D**,**E**,**G**,**H**) High-resolution magnetic resonance images obtained in a spatial sequence in the axial T2 AX (TR/TE: 4500/84) and in the sagittal PD FS (TR/TE: 3805/36) plane were used to evaluate the treated osteochondral defects six months after surgery. The arrow indicates the operated area. (**A**,**B**) In the αTCP + M25 biocement group, the defect was completely filled with newly formed tissue. (**D**,**E**) In contrast, in the spontaneous healing group, the osteochondral defect was not completely restored by newly formed tissue, the cartilage surface was clearly reduced compared to the surrounding native cartilage, and a clear interface between the original and regenerated tissue was visible. (**G**,**H**) For comparison, axial and sagittal images of healthy cartilage are also included. Using standard X-rays, we achieved the required spatial resolution in the examined knee joints. (**C**) In the αTCP + M25 biocement group, we recorded uniform, translucent narrowing of the joint space on lateral X-rays, no pathological structures were observed in the knee joint cavity. (**F**) X-ray examination in the experimental group after spontaneous healing demonstrated uniform size of the joint space, (**I**) compared to the X-ray examination of a healthy knee joint.

Conventional radiographic examination (X-ray) and magnetic resonance imaging (MRI) were performed to evaluate the treated osteochondral defects. In the animal group with honey biocement, lateral radiographs demonstrated a preserved and evenly distributed joint space without evidence of osteophyte formation (Figure 16C) or deformities affecting the femur, femoral condyles, or tibia. Plain radiography enabled the assessment of structural changes in the adjacent subchondral bone, including the presence of subchondral osteosclerosis or cystic lesions. In this first group of animals, no pathological alterations indicative of progressive joint degeneration were observed. In the biocement-treated group, no pathological radiographic changes were detected. Radiographic evaluation in animal group with spontaneous healing process similarly demonstrated a uniform joint space between the epiphyses, without pathological alterations in the surrounding soft tissues (Figure 16F), compared to the X-ray examination of a healthy knee joint (Figure 16I).

MRI assessment of defects treated with biocement revealed a smooth articular surface of the neocartilage with a homogeneous internal structure and complete integration with the surrounding native articular tissue. Detailed cross-sectional analysis of the osteochondral defect confirmed complete regeneration of both cartilage and subchondral bone in the biocement group, with the thickness of the regenerated cartilage measuring approximately 1.5 mm.

Furthermore, in all defects treated with biocement, the measured signal intensity was comparable to that of the adjacent native articular cartilage. Complete integration of the newly formed subchondral bone with the surrounding native subchondral bone tissue was observed.

Detailed MRI analysis following treatment with honey biocement demonstrated 100% defect filling, with the reparative tissue occupying the entire defect area. Integration with the surrounding osseous and subchondral tissues was complete, and the boundary between the regenerated and native tissues was only faintly discernible. The newly formed subchondral bone exhibited a homogeneous structure (Figure 16A,B).

The thickness of the covering cartilage at the level of the defect was uniform, without apparent thinning. The surface contour of the regenerated cartilage showed no significant irregularities or newly formed minor defects compared to the adjacent healthy cartilage. The internal structure of the repair cartilage at the defect site was only minimally inhomogeneous.

In terms of signal characteristics, the covering cartilage at the defect level demonstrated a subtle hyposignal on T1-weighted images and was nearly isointense on the proton density fat-suppressed (PDFS) sequence relative to the surrounding cartilage.

MRI examination demonstrated that, following the creation of an osteochondral defect on the medial femoral condyle and implantation of honey biocement, sclerotic changes were present circumferentially around the defect, indicating an ongoing healing process. Signs of cartilage regeneration were observed at the defect site, with the thickness, structure, and signal intensity of the repair tissue comparable to those of healthy native cartilage (Figure 16G,H).

These findings confirm that the healing process in knees treated with honey biocement was enhanced compared to the animal group with spontaneous healing six months after implantation. The signal characteristics of the regenerated tissue were comparable to those observed in control knees.

In contrast, the group of spontaneous healing exhibited a rough articular surface with shallow superficial ulcerations and morphological irregularities, including fraying, fissuring, and fibrillation, without exposure of the subchondral bone. MRI evaluation in this group revealed incomplete healing six months postoperatively, with neo tissue formation reduced to approximately 50% of the original defect volume (Figure 16D,E). The thickness of the regenerated cartilage was detectable on MRI predominantly at the periphery of the defect, while the central region demonstrated a reduction in chondral tissue of up to 50%.

Tissue analysis further characterized the incomplete repair by low signal intensity in the central portion of the defect, confirming insufficient structural restoration.

## 4. Discussion

The management of osteochondral defects (OCDs) continues to represent a significant challenge in contemporary orthopedic research, as the restoration of both articular cartilage and the underlying subchondral bone is biologically and mechanically demanding. Preclinical animal models play a crucial role in evaluating regenerative strategies and assessing their translational potential prior to clinical application [57]. These models allow for detailed investigation of biological responses, cartilage repair processes, subchondral bone remodeling, and the functional integration of newly developed therapeutic approaches. Experimental models in small animals (rats, rabbits) are frequently used for biomaterial screening and the evaluation of osteochondral regeneration; however, their limitations include differences in joint biomechanics and the spontaneous regenerative capacity of cartilage, particularly in rabbits [58]. In contrast, large animal models (sheep, goats) more closely resemble the biomechanical and histological properties of human articular cartilage, thereby increasing their translational value [57] Nevertheless, none of the a forementioned models can completely reproduce the complex pathophysiology of osteoarthritis or chronic osteochondral lesions in humans. Among large animal models, the pig demonstrates the greatest anatomical similarity to humans, both at the ultrastructural level and in terms of measurable biomechanical characteristics. The pig represents a highly relevant large animal model in orthopedic research due to several anatomical, biomechanical, and histological similarities to humans. Its advantages include the comparable size and morphology of the knee joint, as well as similar biomechanics of the weight-bearing surface and overall body mass [59]. Importantly, pigs lack the capacity for spontaneous healing of larger osteochondral defects, which allows for a more clinically relevant assessment of regenerative strategies. Furthermore, the porcine model demonstrates comparable trabecular bone thickness and a lamellar bone structure closely resembling that of humans [60]. Additional similarities include the rate of appositional bone growth, the organization of the collagen network within the extracellular matrix [61], and the thickness of articular cartilage.

In recent years, research has increasingly focused on cell-based therapies, biomaterial scaffolds, composite osteochondral constructs, and bioactive molecules designed to improve structural organization and long-term tissue stability [4,62,63]. Particular attention has been devoted to the role of subchondral bone in osteochondral repair, as alterations in this compartment significantly influence the quality and durability of regenerated cartilage tissue [64,65]. Collectively, these findings highlight the necessity of biologically driven and structurally optimized treatment strategies aimed at achieving durable and functionally relevant restoration of osteochondral lesions.

Our results highlight the remarkable properties of α-tricalcium phosphate (α-TCP) in the investigated samples, as calcium phosphate-based biomaterials are well known for their osteoconductivity, bioresorbability, and ability to undergo in situ hydrolysis into calcium-deficient hydroxyapatite, thereby providing structural support and promoting osteochondral tissue regeneration [33,66]. Their injectability and moldability enabled adaptation to irregular defect geometries, which is particularly advantageous in the treatment of osteochondral lesions.

Current literature offers only limited insight into the effect of honey on the material properties of calcium phosphate biocements. Following the incorporation of Manuka honey (M25), the regenerative microenvironment appeared to be further enhanced due to its well-documented antibacterial, anti-inflammatory, and wound-healing properties [45,67]. The high methylglyoxal content contributes to its antimicrobial activity, while its immunomodulatory effects may promote early tissue repair and remodeling. Modulation of the inflammatory microenvironment is particularly critical in osteochondral repair, as excessive or prolonged inflammatory responses may negatively affect chondrogenesis and subchondral bone regeneration [66]. In vitro cytotoxicity assays confirmed the non-cytotoxicity of honey–cement extracts. Honey incorporation enhanced alkaline phosphatase activity, promoted calcium deposition, and upregulated osteogenic markers (osteopontin, osteocalcin, and osteonectin) in mesenchymal stem cells. Collectively, these results demonstrate a synergistic effect between honey and CPCs, leading to enhanced bioactivity and supporting their potential for bone regeneration applications [37,40,68,69].

From a biomechanical perspective, the combination of a bioactive component (Manuka honey) and a calcium phosphate biocement (α-tricalcium phosphate) positively influenced porosity, degradation kinetics, and local ion release, thereby modulating the cellular response as well as the osteogenic and chondrogenic potential of the biomaterial [70]. The controlled degradation of α-TCP supports gradual load transfer to newly formed bone, which is particularly important in weight-bearing regions. However, the optimal concentration of honey must be carefully determined, as excessive incorporation may adversely affect mechanical strength or interfere with the cement setting process.

In the therapeutic application of Manuka honey in combination with α-TCP for the treatment of osteochondral defects, particular attention must be paid to its concentration, as this determines its biological activity. Its biological effects are closely associated with the content of methylglyoxal and other reactive components, whose activity is strongly dose-dependent [71,72].

At lower concentrations Manuka honey promotes favorable modulation of the inflammatory response and stimulates tissue repair processes. It has been demonstrated that honey can influence the activity of immune cells, including macrophages, and enhance the production of growth factors that are essential for angiogenesis and tissue regeneration [39]. In the context of osteochondral defect therapy, such immunomodulation creates a microenvironment conducive to the migration of mesenchymal stem cells and their differentiation toward osteogenic and chondrogenic lineages. These findings are consistent with the outcomes of our study, which demonstrated the formation of hyaline cartilage at the site of the original defect in the porcine knee joint.

Conversely, higher concentrations of Manuka honey may adversely affect the overall therapeutic process due to potential undesirable effects, including increased generation of reactive oxygen species, reduced cell proliferation, and possible cytotoxic reactions. In vitro studies have shown that elevated levels of methylglyoxal can negatively influence cell viability and extracellular matrix production [73,74]. Under osteochondral defect conditions, where diffusion is limited and local concentrations of bioactive compounds may accumulate, careful dose optimization is therefore essential. This aspect also played a crucial role in the design and interpretation of our study.

The outcome of an experiment in which the application of Manuka honey in combination with other bioactive substances plays a significant role is fundamentally influenced by the appropriate selection of the animal model. Recent review studies emphasize that small animal models (rats, rabbits) exhibit a higher intrinsic regenerative capacity of cartilage and distinct biomechanical characteristics, which may lead to an overestimation of the therapeutic efficacy of biomaterials and an underestimation of their potential adverse effects [75]. Faster metabolism and thinner cartilage may mask dose-dependent cytotoxic effects of bioactive components. In contrast, large animal models, particularly the pig, provide biomechanically and histologically more relevant conditions for the evaluation of osteochondral regeneration. The porcine knee joint is characterized by comparable articular cartilage thickness, similar subchondral architecture, and physiological loading conditions close to those of the human joint, thereby enabling a more reliable assessment of long-term implant integration, chronic inflammatory response, and mechanical stability of the regenerated tissue [76]. From a translational perspective, large animal models are therefore particularly essential when evaluating biomaterials containing biologically active substances with potentially dose-dependent effects.

Clinical studies of the dynamics of biochemical indicators that characterize the reactive response and regenerative processes in the organism are of scientific and practical importance. Elevated levels of total protein (TP) and globulins (GLOB) observed in stages III–V indicate hyperproteinemia associated with immune activation and increased collagen synthesis during tissue healing [77,78] (Albeshri et al., 2018; Hess, 2005). The decreased A/G ratio further suggests the presence of inflammation, which is typical of early regenerative phases but may impair tissue remodeling if persistent [79,80].

Increased BUN levels, together with transient changes in ALT and AST, reflect enhanced metabolic activity and protein catabolism during active tissue repair, without evidence of permanent tissue damage [79,81,82,83]. Decreased alkaline phosphatase (ALP) activity and lower phosphorus (PHOS) levels indicate delayed mineralization and reduced osteoblastic activity, potentially associated with inflammation or oxidative stress [84,85].

In contrast, stable levels of GGT, glucose, and cholesterol suggest preserved metabolic homeostasis without systemic toxicity. Overall, these findings reflect a transition from an initial inflammatory phase toward progressive tissue regeneration and restoration of bone homeostasis.

The monitoring of targeted metabolites provided additional insight into tissue regeneration and graft integration processes. Increased hydroxyproline levels in stages III and IV indicate enhanced collagen synthesis and extracellular matrix remodeling, which are essential for tissue healing and bone regeneration [85].

Decreased concentrations of creatinine, tyrosine, tryptophan, and adenosine in stages II–IV suggest alterations in metabolism and immune response following implantation. Lower amino acid levels may reflect their increased utilization for protein synthesis or temporary suppression of immune activity. Reduced adenosine levels may also indicate an imbalance between pro-inflammatory and anti-inflammatory mechanisms during early healing phases [86].

An increase in neopterin in later stages indicates activation of the immune system, associated with both regenerative processes and the response to the graft [87]. Methionine and hypoxanthine levels remained relatively stable, while a transient increase in methionine in early stages may be linked to oxidative stress [88].

Overall, these changes reflect the transition from an inflammatory phase to active tissue remodeling and progressive regeneration.

The aim of the study by Yatsun et al. [89] was to monitor blood biochemical parameters after the application of bioinert and biodegradable tibial implants based on the magnesium alloy MA-10. During the study, it was found that significant (*p* ≤ 0.05) differences in enzyme activity indicators occurred only before and immediately after surgery. This can be explained by the stressogenic effect of the injury rather than by the contact of the implant with the tissue. The total protein (TP) content in both groups decreased significantly only immediately after surgery, which is clearly associated with blood loss. No deviations were observed in the subsequent observation periods. The results of creatinine and total bilirubin levels in plasma showed that the maximum values are associated with the acute phase of the pathological process. No significant fluctuations in glucose levels were observed in any of the monitored groups. The results of our analyses correlate with the values described in the experiment.

In another study [90], basic biochemical parameters of blood serum in laboratory rats were evaluated after filling a defect in the metaphysis of the femur with allogeneic bone implants. It was found that alkaline phosphatase (ALP) activity was lower 14 days after implantation, similarly to our results, which indicates a slowdown in bone formation processes, namely a decrease in osteoblast activity.

Future research on osteochondral lesion treatment should focus on optimizing scaffold design to more closely replicate the native architecture of cartilage and subchondral bone while preserving their functional and mechanical properties. Despite the promising outcomes associated with scaffolds incorporating bone marrow–derived cells, growth factors, and cytokines, their broader clinical application remains constrained by several limitations, including complex storage requirements, reduced cell viability, and the instability of bioactive molecules. These challenges may compromise reproducibility and limit the long-term efficacy of such therapeutic approaches.

Furthermore, the complexity of biologically active scaffold systems poses additional challenges in terms of standardization and regulatory approval, potentially hindering their translation into routine clinical practice. In contrast, cell-free scaffolds offer a simpler and potentially more reliable alternative; however, their ability to fully recapitulate the biological activity provided by cells and signaling molecules remains uncertain. Therefore, future strategies should aim to achieve a balanced approach that integrates the advantages of both systems while minimizing their inherent limitations.

## 5. Conclusions

The combination of a calcium phosphate-based biocement with Manuka honey was successfully applied for the treatment of osteochondral defects in the knee joints of domestic pigs, resulting in complete defect filling with smooth, homogeneous tissue exhibiting native hyaline cartilage characteristics six months post-surgery. These findings suggest that the regenerative success is driven by the synergistic action between the osteoconductive biocement and the bioactive constituents of Manuka honey, specifically methylglyoxal (MGO) and phenolic derivatives. Consequently, these results support the potential of this composite as a promising regenerative strategy, where Manuka honey serves not merely as an antibacterial agent but as a primary bioactive modulator of the structural, biological, and biomechanical aspects of osteochondral healing.

The combination of biocement and honey significantly promoted osteochondral tissue regeneration. The observed outcomes were comparable to those of native healthy cartilage.

## Data Availability

Data is contained within this article.
